# Strategic and self-regulated instruction in synthesis tasks in the university context

**DOI:** 10.3389/fpsyg.2024.1386907

**Published:** 2024-07-02

**Authors:** P. Robledo, M. L. Álvarez

**Affiliations:** Department of Psychology, Sociology and Philosophy, University of León, León, Spain

**Keywords:** reading strategies, strategic and self-regulated instruction, synthesis task, text quality, university, writing strategies

## Abstract

**Introduction:**

The aim of the study was to improve student skills in writing good-quality synthesis texts through a strategic, self-regulated instruction program aimed at ensuring that students properly activated reading and writing strategies required by the synthesis task.

**Methods:**

The sample consisted of 84 university students who were randomly assigned to experimental or control conditions. The experimental group received an instructional program based on the development and self-regulated implementation of reading and writing strategies for producing synthesis texts. The control group received a program involving metacognitive knowledge of various academic text types. Both programs involved eight 60-min sessions, taught by teachers in a compulsory degree subject. For the evaluations, students produced synthesis texts from different source texts. The syntheses were graded considering text product measures: information selection, idea connection, text organization, and holistic quality; and measures of reading (underlining and note-taking) and writing (planning and review) strategies.

**Results:**

The results show that the experimental group exhibited greater improvements in synthesis quality and greater improvements in activation of information organization processes, note-taking while reading, and text planning.

**Discussion:**

In conclusion, university students can, following implementation of a strategic instructional procedure in the context of a study plan, adapt and re-work their own reading and writing strategies and apply them in a self-regulated manner to synthesis tasks, improving text quality and some of the cognitive processes involved.

## 1 Introduction

Nowadays in higher education, students are often expected to demonstrate their communicative competency when given tasks in which they have to manage information from various sources and produce written work based on that information (Boscolo et al., 2007; Roldán et al., 2011). These tasks follow the typical format of synthesis tasks (ST), which teachers routinely ask their students to do in the classroom as part of their teaching-learning activities (Perin, 2013).

STs are hybrid tasks that require students to use a combination of reading comprehension skills and writing skills (Granello, 2001; Solé et al., 2005, 2013). They involve analysing different sources of information about a topic and producing a new text from that information, appropriately comparing, transforming, and combining ideas in an organized way. Students have to select information, connect it, and organize it in a new text structure to produce new, original written discourse (Spivey and King, 1989; Spivey, 1997). Successfully completing STs demands selecting, organizing, and connecting information. Students must select information because they cannot retain all of the information from what they read, they must choose information to deal with based on relevance or importance. They also have to organize what they have selected within some structure in order to construct a mental representation of the texts, and they must connect the information at a local level to form a new cohesive, coherent discourse (Spivey and King, 1989). STs require students to use recursive reading and writing strategies (Flower et al., 1990; Spivey, 1997; Segev-Miller, 2004, 2007; Boscolo et al., 2007; Mateos and Solé, 2009; Solé et al., 2013). They need to read and re-read the source texts and their own writing, re-examining the source texts to identify relevant information and incorporate it, and re-examining their own text to correct it in a recurrent process allowing the author to transform the source information (Segev-Miller, 2004). ST is also a demanding writing task requiring the mediation of planning, textualization, and monitoring throughout the whole process (Flower and Hayes, 1980), as well as the use of review strategies ensuring a good fit between the student’s on text and the source texts. Previous studies have demonstrated differences in the reading and writing strategies used by students with different levels of expertise in STs, as well as a certain correspondence between the holistic quality of the written product and the strategies used. They also indicate that the more successful ST products are associated with using a range of strategies, and that failed texts are associated with students adopting simple, direct, linear processes that are inadequate for the complexity of STs (Solé et al., 2005, 2013; Mateos and Solé, 2009; Du and List, 2020; Nadal et al., 2021).

The inherent cognitive complexity of STs means that they are highly demanding tasks at the level of cognitive processing and activity, which leads to some university students finding them difficult. Some students approach writing STs in a linear, fixed manner, limiting themselves to repeating the content from the sources and not incorporating or properly organizing the information, as well as applying strategic approaches that are less than effective (Boscolo et al., 2007; Mateos and Solé, 2009; Cumming et al., 2016). These students seem to need specific training that will enable them to do synthesis tasks, and there is empirical evidence of how guiding students to perform these types of tasks produces very satisfactory results. Some studies have confirmed the efficacy of ST instruction in the university context. For example, Zhang (2013) examined the effect of instruction in ST in 190 students split into a control and an experimental group. In the experimental group, five iterations of instruction about writing syntheses were incorporated into the existing course curriculum, which was built on reading, writing, and vocabulary instruction from two textbooks. The students in the experimental group were guided through the discourse synthesis writing process and a range of methods such as reading guides, connection exercises, and peer and teacher feedback on first drafts were used to scaffold the students’ synthesis writing practice. The control group had an equivalent amount of reading and writing practice in other writing types within the same curriculum. The experimental group had significantly better performance in writing synthesis texts, and the study also confirmed the feasibility of incorporating ST instruction in the classroom without significantly altering the study plan, albeit with a notable amount of scaffolding by teachers, materials or peer support. Boscolo et al. (2007) performed an intervention in which 52 students were asked to analyse the characteristics of good and poor synthesis texts, and to write and revise their own. The intervention included 10 sections, with both theoretical lectures on the writing models and practical workshops. Participants practiced academic writing by composing texts which were revised by the teacher, and by analysing and discussing examples of good and poor academic texts. The results showed an improvement in the students’ abilities to write synthesis texts. The intervention was mostly focused on analysis of text characteristics and had an impact on the students’ ability to select information based on structural cues in the text. However, the intervention did not affect the students’ ability to integrate this information in a coherent representation and text structure, probably because it did not include training in procedural or strategic knowledge.

In the Spanish context some studies have included strategic or procedural training. For example, Mateos et al. (2018) evaluated the effectiveness of two types of interventions aimed at improving performance in synthesis tasks, including procedural training. One used a graphical guide with peer practice, the other added explicit instruction with modelling and explanation of the processes involved in synthesis tasks (procedural training). The results showed that the students who had the additional explicit instruction demonstrated substantial improvements in the quality of their work. Granado-Peinado et al. (2019) analysed the impact of interventions involving collaborative writing of argumentative syntheses, which combined explicit instruction with video modelling of writing and collaboration processes, along with a guide and collaborative practice. The effects of this intervention were compared with three other programs in which the amount of help was progressively reduced. Their results showed that in order for students to achieve the desired levels of competency, interventions should include explicit instruction with video modelling. When this instruction brings together help aimed at improving production of argumentative syntheses with help designed to promote collaboration, students can assimilate a greater amount and wider range of information. However, for identifying a high level of arguments, explicit instruction focused solely on helping students write argumentative syntheses turns out to be as effective as help aimed at collaboration. In addition, after interventions encouraging collaborative work, students successfully transfer their newly developed skills to their own, individual writing tasks. In both studies, teachers demonstrated how to perform ST, teaching specific procedures and strategies. Students usually learn to emulate these procedures but do not produce their own strategies.

All of these previous studies have demonstrated the efficacy of instruction in ST in the university context and included content or components of strategic, self-regulated instruction, such as declarative or procedural metacognitive knowledge linked to the text product or the writing process, the use of cognitive strategies, direct teaching about procedural and discourse knowledge writing strategies, modelling, and collaborative or individual practice. The strategic instructional approach has been shown to be one of the most effective ways to improve students’ writing competence at various educational levels (McArthur and Philippakos, 2013, 2022; Graham et al., 2018; Graham and Harris, 2018; van Ockenburg et al., 2019), including university (McArthur et al., 2015, 2022). That said, its complex, multicomponent nature makes it difficult for teachers to use in the real university classroom, it needs a lot of time to implement, along with specific teacher training that normally requires professional development programs (Harris et al., 2022). Furthermore, implementation of strategic instruction programs aimed at encouraging mastery of ST at the university level must consider the characteristics of the students, who usually exhibit relatively defined or mature strategic profiles (Kieft et al., 2007; Robledo et al., 2018; Arias-Gundín and Robledo, 2023). This means that rather than focusing on giving students specific strategies to produce their syntheses, instruction in ST at university should focus on guiding and prompting students so that, as part of the natural learning process in their subjects, they develop and implement their own reading and writing strategies in a self-regulated manner when doing ST. This would allow them to manage their cognitive and motivational processes and keep on with a task even if they become discouraged or find it difficult, which would contribute to a successful approach and consequent learning or mastery (Butler, 1998; van Ockenburg et al., 2019). This is exactly where the present study sits, with the aim of assessing whether it is possible to improve university students’ competencies for synthesis tasks, via strategic instructional processes which, as part of the context of the university subjects, promote self-regulated application of reading and writing strategies by the students. A line that, as far as we are aware, previous research has not addressed.

### 1.1 Present study

The study looked at an intervention to improve university students’ ability to compose good-quality synthesis texts. The intervention implemented a program of strategic and self-regulated instruction so that students would use suitable reading and writing strategies consistent with the demands of synthesis tasks. The instruction was based on the Strategy Content Learning model (SCL, Butler, 1992) theoretically based on self-regulated learning, which is crucial for students’ academic performance in all education levels (Alonso-Tapia, 2012; Rosário et al., 2017). According to that model, when students face an academic task, they enter a recursive cycle of cognitive and metacognitive activities that involve analysing the demands of the task, selecting, adapting, and employing suitable strategic approaches, monitoring the application of these strategies, producing feedback, and readjusting (see revision in Robledo and García, 2017). In the context of written work specifically, the SCL model has proven effective when applied to students in further and higher education. Students improve the quality of their compositions, increase their metacognitive knowledge about this kind of writing, and improve their ability to adapt and effectively activate their own writing strategies for various writing tasks, taking a self-regulated approach to composing texts. They also have an improved sense of self-efficacy and they improve their attributional patterns (Butler, 1994, 1995, 1996, 1997, 1998).

For the present study, the experimental group were instructed according to the SCL model through a program focused on promoting self-regulated application of their own reading and writing strategies to the production of a synthesis text. The study also included a control group, who received instruction on metacognitive knowledge around different types of academic texts at university, including synthesis. The question the study addresses is whether the students in the experimental condition—instructed strategically according to the SCL model—effectively adapt and activate their reading and writing strategies in the production of synthesis texts, and consequently produce better quality texts than the control group. Based on the evidence about the efficacy of strategic, self-regulated instruction in the general field of writing (Graham et al., 2018; Graham and Harris, 2018), about the SCL model in particular (Butler, 1994, 1995, 1996, 1997, 1998), and about university students’ instruction in synthesis tasks (Boscolo et al., 2007; Zhang, 2013; Mateos et al., 2018; Granado-Peinado et al., 2019), our initial hypothesis is that we expect an increase in the effective activation of the reading and writing strategies involved in producing synthesis texts, as well as an improvement in the quality of the written product from the students in the experimental group compared to the control. In other words, we anticipate that the students in the experimental group will produce or adapt their own reading and writing strategies to the execution of synthesis tasks so that they improve their performance at the level of the written product, producing better quality syntheses than the control group.

## 2 Methodology

### 2.1 Sample

The sample comprised 84 students who were doing a degree in Early-childhood Education at a Spanish university. They were aged between 18 and 35 years old (M = 20.18 years, SD = 2.74). Just over a tenth (13.1%) were men, 86.9% were women (χ^2^_(4, *N* = 84)_ = 85.058, *p* < 0.001). The students were selected through a non-probabilistic sampling process from four class groups in an obligatory subject in the first year of the degree (group 1, *n* = 21; group 2, *n* = 20; group 3, *n* = 22; group 4, *n* = 21). Two class groups were randomly assigned to the control condition (groups 1 and 2, *n* = 41, 12.2% men) and two to the experimental condition (groups 3 and 4, *n* = 43, 13.9% men). The experimental group received the program of strategic, self-regulated instruction on synthesis tasks. The control group received a program of instruction based on developing their metacognitive knowledge of the structure of various types of scientific or academic texts, including synthesis.

### 2.2 Measures

At each assessment timepoint (pre-test, post-test, and follow-up) students were given a writing task consisting of producing a synthesis text. The evaluator explained to the students that they had to compose a synthesis text after reading two source texts. The source texts were given to the students by the evaluator and dealt with the corresponding topic in each task. The synthesis text could be a maximum of two pages long. The tasks dealt with various topics (e.g., Metacognition, Self-concept, ICT and education, and Leisure and free time), counterbalanced by condition and timepoint. The source texts were created by the researchers using various psychology and education manuals, ensuring similar characteristics in terms of length (870–970 words), structure (the same number of paragraphs), and content (25–30 main ideas and 6–8 topic units, in both cases identified by three researchers independently). The students were asked to write the best synthesis text possible. They were given a blank draft page to use if they chose, and a blank sheet for their final text. The students were also free to markup the source texts as they wished (underline, annotate, highlight, etc.). There was no time limit for the tasks.

Each task was used to assess students’ writing competence with two types of measures (see Table 1). The first type were text product measures, assessed via the synthesis texts themselves: selection of information, connecting ideas, text organization, and holistic quality of the text. These were assessed blind by two reviewers who had been trained beforehand, with a kappa inter-rater index over 0.70 in each of the dimensions analysed (Selection of information: 0.83, Connection: 0.71, Organization: 0.88, Quality: 0.70). The other type of measure was reading and writing strategies measures. Reading strategies, underlining and note-taking, were assessed by looking at how the students treated the source texts. In this case, a reviewer analysed the students’ source texts and identified the number of underlined texts (none, one or two texts underlined), the type of underlining (undifferentiated: same colour, format; or differentiated: different colour, format), the number of texts with notes (0, one text or two text with notes), and the type of notes (without elaboration: only one word or symbol; with elaboration: sentences, arrows joining content sentences, explanations). Writing strategies, planning and revising, were assessed by examining the students’ drafts (planning strategies) and final texts (revising strategies). For the planning strategies, the reviewer analyzed how students used the draft sheet to plan their synthesis texts, evaluating that based on the parameters given in Table 1. For the revising strategies, two reviewers examined the final synthesis text and identified whether corrections had been made or not, and if there were corrections, whether they were mechanical (spelling, handwriting, grammar) or substantive (adding, changing, deleting, reordering or adding to ideas, etc.).

**Table 1:** Measures of students’ writing competence.

Measure	Scale
**Product measures**	
**Selection of information**	
1.1. Idea selection: Main ideas (MI) from source texts appearing in the synthesis	0 MI from only one source text 1 Only MI common to both texts 2 Almost all MI, both common to both texts and otherwise 3 All MI
1.2. Informative capacity: Topic units (TU) from source texts appearing in the synthesis	0 Lacking any TU 1 Less than half of the TU 2 Half of the TU 3 More than half of the TU 4 All of the TU
**Connecting ideas**	
2.1. Cohesion: incorporation of ideas from the source texts in the synthesis	0 Two separate summary texts of the sources juxtaposed. 1 Alternating ideas from the two sources, using connectors, subordinate phrases, etc. 2 Presents its own structure and incorporates ideas from both texts in new discursive units, combines ideas.
2.2. Creation-plagiarism: level of elaboration present in ideas in the synthesis	0 Literal copy of ideas in the source texts 1 Copies some ideas, paraphrases others 2 Copies and paraphrases, but also has some elaboration 3 Demonstrates elaboration
**Text organization**	
Overall structure of text: has an introduction, paragraphs, conclusion. Displays hierarchy	1 point each for presence of: Introduction: summarizes, anticipates or presents content Paragraphs: the information is organized in paragraphs Conclusions: the writer’s opinion, final considerations. Hierarchy: organization of information according to importance and reciprocal relationships
Local structure: cohesion of paragraphs and ideas through discourse markers	Number of indicators: meta-structural, structural, reformulating, and argumentative.
**Holistic quality**	Rate the quality of the synthesis (1 low, 5 high) based on indicators of: introduction, organized details, fluid discourse, clear sequence of ideas, no digression, structural markers, conclusion, vocabulary, syntax, spelling, and presentation.
**Reading strategy measures**	
**Underlining**	
Underlining	0 Not present 1 Undifferentiated 2 Differentiated
N° of underlined texts	0 None of the source texts underlined 1 One source text underlined 2 Both source texts underlined
**Note-taking**	
Type of notes	0 No notes taken 1 Notes without elaboration 2 Notes with elaboration
N° of texts with notes	0 No notes on either text 1 Notes on one text 2 Notes on both texts
**General reading strategy**	0 Text is un-annotated 1 Only underlining 2 Only notes 3 Underlining and notes
**Strategy measures**	
**Planning**	
Overall planning	The level of general planning activity shown by the draft 0 No draft 1 Draft poorly developed 2 Draft partly developed 3 Draft well-developed
Planning ideas	How the writer produces and organizes ideas: 0 Ideas developed in the text 1 Ideas organized according to a plan or structure 2 Ideas in a list, with no order, apparently unconnected
8.3. Type of planning	Allusion to or presence in the draft of: introduction, development, conclusion, objective, audience, and strategy. 0 No draft 1 No reference to this aspect 2 Graphical reference to this aspect (list, word) 3 Explicit allusion to this aspect, written or drafted
**Revision**	Number of effective revisions shown in the synthesis: mechanical, substantive, total

Measures collected in the “Synthesis assessment protocol,” produced by the authors’ team of experts on writing competency, following review and adaptation of various of their own tools and those from the scientific literature (Spivey and King, 1989; Boscolo et al., 2007; Martínez et al., 2011).

### 2.3 Design and procedure

The study followed an experimental pre-test, post-test, follow-up design with a control group. The independent variable was the instruction condition (experimental-control) and the dependent variables were the reading and writing strategy measures and the text product measures. The students gave their informed consent and were then randomly assigned to each condition. The experimental condition was given the strategic program (summarized in Table 2), designed based on SCL. This followed a sequence of three phases in which the students, guided by the instructor, worked on identification, development, and effective, self-regulated application of their strategies. In the first phase, the instructor offered support and assistance to the students so that they could analyse the task, identify objectives, and define criteria for correctly completing it. In the second phase, the instructor guided students so that, starting with identifying their own strategies and analysing their effectiveness, they developed suitable strategic approaches to the task. In the final phase, the instructor supported the students while they applied their strategies, encouraging monitoring and guiding them in self-feedback, finally suggesting strategic reconsideration.

**Table 2:** Instructional program experimental condition.

Sequence	Focus	Instructor	Activities
Phase 1	Session 1 (60 min. February, week 2). Developing metacognitive knowledge of ST and its demands (product and strategies)	Teacher 1: group 3 Teacher 2: group 4	After producing an ST (pre-test): Students describe what they felt the task demanded and the strategies they used. They answer two questions: What should you do? How did you do it? ST analysis by the teacher, examining the true product demands and activation of reading-writing strategies and processes. The teacher explains: What are synthesis tasks. What are their characteristics in terms of selection, connection, and organization processes. The demands of ST at the level of writing and reading processes The teacher presents a self-monitoring sheet to assess the quality of the syntheses at the product level Pool ideas about ST and strategic procedures used by students in the pre-test ST.
	Session 2 (60 min. February, week 4). Set suitable goals for doing STs	Teacher 1: group 4 Teacher 2: group 3	Students set individual goals related to STs in terms of product and the cognitive processes required. Using the self-monitoring sheet from session 1 as a starting point, students make their own self-monitoring sheet; it includes the individual goals about product quality and assessing it to facilitate self-regulation for the process of producing STs. Pool ideas
Phase 2	Session 3 (60 min. March, week 2). Develop understanding of strategies for producing synthesis and examine efficacy of students’ own strategies	Teacher 1: group 3 Teacher 2: group 4	The teacher analyses and explains examples of effective strategies for synthesis. The teacher presents correct ST analysis, looking at product quality and the analysis of strategic processes used to do synthesis using the self-monitoring sheet, to which they add assessment of the activated reading and writing processes and strategies. As a group, students and teachers recognize effective and ineffective aspects of students’ own strategies (compare model ST and procedure and students’ own STs).
	Session 4 (60 min. March, week 4). Define students’ own strategies	Teacher 1: group 4 Teacher 2: group 3	Students reflect on their (pre-test) STs and examine whether they met the criteria of a quality synthesis according to the teachers’ self-monitoring sheet. They identify what fell short in the product and activation of reading and writing strategies and processes. Students set individual and definitive goals related to STs in terms of product and the cognitive processes required. Students define their own strategies and adjust their own self-monitoring sheet, with the teacher’s help. Pool ideas.
Phase 3	Session 5 (60 min. April, week 2). Learn types of feedback and how to apply them. Produce self-reinforcement.	Teacher 1: group 3 Teacher 2: group 4	The teacher presents types of feedback and models applying them and strategies to STs. Students define self-reinforcement.
	Session 6 (60 min. April, week 4). Apply students’ own strategies to peer texts. Evaluate their efficacy and redefine them.	Teacher 1: group 4 Teacher 2: group 3	Students do an ST task in pairs: One student does the ST task applying the strategies and thinking aloud. The other follows the whole process of creating the synthesis, playing an active role and participating in the process, analyzing possible mistakes and offering their own ideas about strategies and help to guide or re-direct the writing process as needed. Students analyse the synthesis applying their own self-monitoring sheets. Individually, students evaluate the efficacy of their own strategies and redefine them according to their weaknesses. Teachers support students during session.
	Session 7 (60 min. May, week 2). Apply students’ own strategies and self-reinforcement to individual texts.	Teacher 1: group 3 Teacher 2: group 4	Students write individual STs, applying their own strategies and self-reinforcement, and evaluate efficacy with the self-monitoring sheet. As a group, students and teachers read and evaluate ST quality.
	Session 8 (60 min. May, week 4). Evaluate strategic efficacy and define it definitively.	Teacher 1: group 4 Teacher 2: group 3	Students compare the ST from session 7 with the pre-test, identifying the differences in the product and in the process and strategies, using the self-monitoring sheets. Final evaluation of students’ own strategies and adjustment. Review and general reflections on generalizing strategies.

The control group also had 8 sessions of intervention about writing which involved working on structural analyses of different academic and scientific text types, including synthesis (see Table 3).

**Table 3:** Summary of the intervention for the control condition.

Session	Focus	Instructor	Activities
1	Identify students’ own ideas about synthesis and its requirements (60 min. February, week 2).	Teacher 1: group 1 Teacher 2: group 2	Students describe perceived requirements of syntheses After producing an ST (pre-test), students analyse the ST based on students’ ideas Teacher explains synthesis and its requirements: what are synthesis tasks and their characteristics in terms of selection, connection, and organization. They also explain the demands of STs at the level of reading and writing processes.
2	Identify the procedure for writing a synthesis and its relationship with the type of representation task (60 min. February, week 4).	Teacher 1: group 2 Teacher 2: group 1	Students describe the procedure for crafting a synthesis (in pre-test). Individual written reflection about the efficacy of the procedure followed, clarify task requirements
3	Analyse text types, developing declarative knowledge: definition, characteristics, structure, and discourse strategies (60 min. March, week 2).	Teacher 1: group 1 Teacher 2: group 2	Individual reading about the document entitled “Los tipos de texto en español” [*Types of texts in Spanish*] and individual identification of: definition, characteristics, internal structure and discourse strategies for descriptive, narrative, explanatory and argumentative texts. Make a content table. Pool ideas as a group
4	Look at different types of text in the university academic-scientific context (60 min. March, week 4).	Teacher 1: group 2 Teacher 2: group 1	Individual reading of document “La escritura académica en la Universidad” [*Academic writing at university*]: students identify and summarize academic discourse characteristics, text types, characteristics Pool ideas together
5–6	Apply knowledge of text structures to a real text in pairs (60 min. April, weeks 2 and 4).	Teacher 1: group 1 Teacher 2: group 2	Review previous content, characteristics of a synthesis Students write a synthesis in pairs. Students do an ST task in pairs: One student produces the ST applying knowledge about this type of task (characteristics, structure, discourse strategies) while thinking aloud. The other student follows the process, playing an active role and participating, analysing possible mistakes, making their own contributions and offering help and redirection to the process as needed. As a group, students and teacher analyse the resulting synthesis, only modifying the structure (session 6)
7	Apply knowledge of synthesis structure and characteristics to a real text individually (60 min. May, week 2).	Teacher 1: group 2 Teacher 2: group 1	The teachers review characteristics of syntheses (presented in session 1) Students write a synthesis individually, applying knowledge about ST characteristics, structure, discourse strategies.
8	Evaluate efficacy of knowledge gained about synthesis (60 min. May, week 4).	Teacher 1: group 1 Teacher 2: group 2	As a group, students and teachers read the syntheses from session 7 and analyse it based on structural characteristics. Students compare the text from session 7 to the pre-test and evaluate the text quality. General review, rate efficacy of the program

Both interventions focused on analysing and producing scientific-academic texts, including synthesis texts, and comprised eight teaching sessions lasting one hour each, held every two weeks during a term. Both had the same number of practices of synthesis tasks: all students had to write two ST about the same content—Motivation and Learning Strategies (content from the degree course the students were doing)—following the same routine, first in pairs and then individually. The interventions were applied by (2) teachers who taught courses in the degree the study took the sample from (a subject in Developmental Psychology which addressed content related to optimizing child development). The teachers had prior experience of instructional research. This meant that they understood the differences between the two treatment conditions and were able to implement them properly.

The sessions were applied in parallel, so that each teacher gave four intervention sessions to the experimental groups and four to the control, each to a different subgroup of students to avoid possible biases related to teachers’ preferences for one program or the other. In addition, the content for each session was detailed in an application protocol that teachers had to follow (see outline in Tables 2, 3). Compliance with the protocol was examined by a non-participating observer to ensure it was faithfully applied in each session. Finally, the sessions required students to do specific practical or applied tasks. These were collected in an individual portfolio and reviewed to verify the proper delivery of the sessions. Thus, all of the students were trained over the same number of sessions, with the same time constraints, the same number of written practices, and the same teacher characteristics. Students were assessed before (pre-test) and immediately after instruction (post-test), and one year later (follow-up). All of the students in the sample attended all of the evaluation and intervention sessions, meaning that there was no missing data.

### 2.4 Data analysis

Data analysis was performed using SPSS software, version 24. The statistics for kurtosis and skewness showed that the text product variables followed a normal distribution while the strategy variables did not (see Table 4). Because of that, for the text product variables, parametric repeated-measure MANOVAS time by condition were performed. To determine the immediate differential effects of the instruction on the text product measures, we performed an analysis of variance with 2 × 2 repeated measures, with time as the repeated measure (pre-/post-test) and the group as inter-subject factor (experimental or control). In addition, to determine whether, a year after the intervention, the students’ situation in the different text product variables had changed from the initial situation (pre-test) or the post-test evaluation, we performed a 3 × 2 repeated measures analysis.

**Table 4:** Kurtosis and skewness.

Dimension	Measure	Pre-test		Post-test		Follow-up	
		Skewness	Kurtosis	Skewness	Kurtosis	Skewness	Kurtosis
Selection of information	Idea selection	0.279	−0.055	0.803	2.767	−0.734	0.279
	Informative capacity	0.317	−1.968	−1.451	3.740	−0.771	0.728
Connecting ideas	Cohesion	−0.540	−1.770	−1.641	6.955	0.111	−0.091
	Plagiarism	0.229	−0.667	0.551	2.583	0.872	0.076
Text organization	Overall structure	0.828	1.860	0.123	−1.191	−1.294	0.291
	Meta-structural	2.011	2.114	1.322	1.317	1.395	1.134
	Structural	1.393	2.776	0.970	0.399	1.238	0.918
	Reformulating	0.369	−0.569	1.231	2.154	0.357	−0.570
	Argumentative	2.636	8.291	1.706	2.983	1.507	2.466
	Local structure	0.777	0.477	1.334	1.551	0.217	−1.205
Quality	Overall quality	0.335	−0.172	0.262	−0.625	0.382	−1.080
Reading strategies	General read. Strateg.	−1.919	1.807	−3.093	7.826	−0.132	−2.073
	Underlining	−3.408	12.091	−2.213	2.996	−1.813	1.344
	N° underlining texts	−7.746	60.000	.	.	.	.
	Type of notes	−0.193	1.448	0.018	−0.084	0.557	−0.992
	N° of texts with notes	−1.382	0.289	−2.725	5.919	0.218	−1.807
Planning	Overall Plan.	−1.458	0.684	−2.518	5.228	0.235	−1.120
	Plan. Ideas	−0.225	−1.034	−0.100	−0.660	0.764	−1.108
	Introduction	.	.	1.195	0.279	−0.569	−0.583
	Development	−1.828	2.407	−1.877	2.645	1.811	1.827
	Conclusion	4.841	22.331	2.011	3.439	.	.
	Audience	.	.	7.483	56.000	6.708	45.000
	Objective	.	.	.	.	6.708	45.000
	Strategies	4.841	22.331	2.098	2.489	0.962	0.430
Revision							
	Mechanical	1.477	1.824	1.409	2.018	1.945	6.202
	Substantive	2.912	10.733	1.209	0.760	2.419	7.475
	Total	2.184	5.596	0.907	0.427	1.184	1.628

For the strategy variables, to analyse the immediate intra-subject effects of the treatments, we performed Wilcoxon’s non-parametric analysis for two related samples independently for the two groups. Inter-subject effects were assessed using the Mann–Whitney U test for independent samples, testing both groups (experimental and control) at pre-test, post-test, and at follow-up.

## 3 Results

### 3.1 ST product measures

Beginning with the 2 × 2 repeated measures analysis, the multivariate tests of variance gave statistically significant results, with a moderate-large effect size, for the measures of text product: overall structure [*F*_(1, 82)_ = 22,093, *p* < 0.001, η^2^ = 0.276], local structure: meta-structuring [F_(1, 82)_ = 12,468, *p* = 0.001, η^2^ = 0.177] and reformulators [F_(1, 82)_ = 7,366, *p* = 0.009, η^2^ = 0.113], and quality [F_(1, 82)_ = 29,847, *p* < 0.001, η^2^ = 0.340]. The multivariate tests of variance in the 3 × 2 repeated measures test produced statistically significant results, with a moderate-large effect size, for the text product measures: overall structure [*F*_(2, 81)_ = 10,561, *p* < 0.001, η^2^ = 0.310], local structure: meta-structuring [F_(2, 81)_ = 6,349, *p* = 0.004, η^2^ = 0.213] and reformulators [F_(2, 81)_ = 3,058, *p* = 0.056, η^2^ = 0.115], and quality [F_(2, 81)_ = 24,647, *p* < 0.001, η^2^ = 0.517]. Table 5 shows the statistically significant results from the tests of inter-subject effects.

**Table 5:** Statistically significant differences between groups in the comparison between pre-test, post-test, and follow-up in the text product measures.

		Experimental						Control						Pre-post			Pre-post-follow		
														Time*condition			Time*condition		
		Pre		Post		Follow-up		Pre		Post		Follow-up		*F*	*P*-value	η^2^	*F*	*p*-value	η^2^
		M	SD	M	SD	M	SD	M	SD	M	SD	M	SD						
Overall structure		2.12	0.59	2.62	0.55	3.65	0.84	2.27	0.45	2.08	0.27	2.92	1.13	3.361	0.072	0.055	6.293	0.016	0.116
Meta-structural		0.21	0.41	0.82	0.79	1.12	1.50	0.08	0.27	0.12	0.32	0.88	1.29	15.351	<0.001	0.209	8.707	0.005	0.154
Reformulating		2.59	1.72	4.68	2.91	5.77	3.46	2.42	1.79	2.58	1.81	4.96	2.97	6.778	0.012	0.105	4.421	0.041	0.084
Quality		1.53	0.74	2.74	0.75	3.88	0.95	1.62	0.57	1.92	0.68	2.57	0.72	4.949	0.030	0.079	18.105	0.000	0.278

As Table 5 shows, the inter-subject effect of the condition shows that the treatment led to greater improvement in the experimental group than the control group. The improvement was in local structure (meta-structuring and reformulators) and in overall synthesis quality. This improvement continued at follow-up and extended to include the general structure dimension. There were no statistically significant differences in the other measures.

### 3.2 Results relating to students’ reading and writing strategies when producing STs

Wilcoxon’s test indicated statistically significant differences between pre-test and post-test in both the experimental and control conditions (see Table 6). In the experimental group, following instruction, the students improved in 3 variables related to activating reading strategies, 5 related to activation of text planning strategies, and 2 related to revising the texts. The control group improved activation of revision in general, and substantive revision in particular, with no statistically significant differences in any other variable.

**Table 6:** Differences between pre- and post-test in reading and writing strategies in each condition.

	Pre-test		Post-test			
	M	SD	M	SD	*Z*	*p*-value
**Experimental**						
General reading strategy	2.56	0.824	2.88	0.478	−1.960	0.050
Type of notes	0.88	0.537	1.35	0.597	−3.578	0.000
N° of texts with notes	1.41	0.821	1.82	0.521	−2.636	0.008
Overall planning	1.53	0.825	1.85	0.436	−2.271	0.023
Planning ideas	1.65	1.098	2.00	0.739	−2.109	0.035
Introduction	1.00	0.000	1.73	0.719	−3.542	0.000
Conclusion	1.04	0.192	1.36	0.549	−2.309	0.021
Strategy	1.15	0.534	1.48	0.870	−2.449	0.014
Mechanic rev.	0.76	0.955	1.74	2.020	−2.935	0.003
Total rev.	2.56	3.518	3.91	3.995	−2.609	0.009
**Control**						
Substantive rev.	0.92	1.521	2.35	2.682	−2.646	0.008
Total rev.	2.15	2.679	4.04	3.736	−2.503	0.012

**Figure 1:**
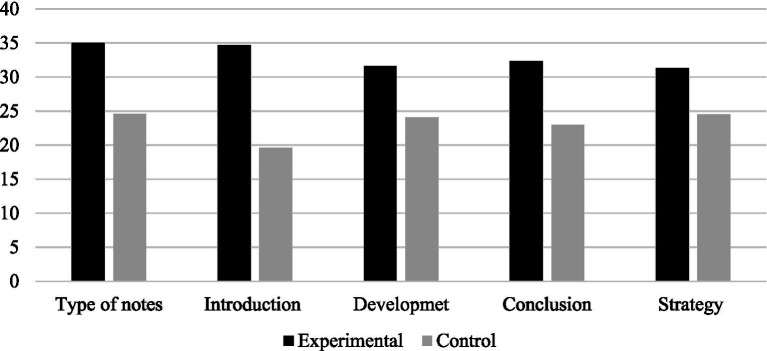
Mean ranks for both groups in the variables with significant differences between condition following the program (post-test).

The Mann-Whitney U test indicated that there were no statistically significant differences between the groups at pre-test for any variable. At post-test there were differences in the type of notes variable (U = 288.000, Z = −2.734 *p* = 0.006) and in planning: introduction (U = 175.000, Z = −4.029 *p* < 0.000), development (U = 277.500, Z = −2.308, *p* = 0.021), conclusion (U = 253.000, Z = −3.055, *p* = 0.002), and strategy (U = 287.528, Z = −2.528, *p* = 0.011). The experimental group had higher (mean rank) scores at post-test, as shown in Figure 1.

At the follow-up timepoint, there were statistically significant differences between the experimental and control groups in overall planning (U = 192.000, Z = −2.648, *p* = 0.008, experimental mean rank score = 30.9 vs. Control mean score = 20.50), Planning: development (U = 158.000, Z = −2.381, *p* = 0.017, experimental mean rank = 26.7 vs. Control mean rank = 18.4), and type of planning (U = 161.500, Z = −2.084, *p* = 0.037, experimental mean rank = 26.54 vs. Control mean rank = 18.6). In each case, the experimental group scored higher than the control.

## 4 Discussion and conclusions

The objective of this study was to improve university students’ competency in composing STs following implementation of a strategic, self-regulated instruction program designed to encourage students to effectively activate the reading and writing strategies demanded by synthesis tasks. To that end, we compared an experimental group, given instruction through a strategic program that was based on Strategy Content Learning (SCL, Butler, 1992), with a control group, who were taught metacognitive knowledge of different text types in the academic context. Our hypothesis, based on the literature in this field, was that compared to the control group, the students in the experimental group would exhibit greater effective activation of their reading and writing strategies during the synthesis tasks and would demonstrate more improvement in their performance in terms of text product, producing better-quality syntheses.

The results confirmed this hypothesis, and with it, the efficacy of the strategic, self-regulated instruction program for promoting improvements in the quality of university students’ synthesis texts, and in the activation of reading and writing strategies. Compared to the control group, the students in the experimental group demonstrated greater improvements in the overall quality of their synthesis texts, producing work with connected, hierarchical ideas and details, demonstrating clear sequences of ideas and fluid discourse. Their texts also exhibited appropriate text organization, with paragraph structures for introductions, development and conclusions that were suitably cohesive. In addition, their texts displayed suitable levels of vocabulary, syntax, spelling, and presentation.

Following the program, the experimental group also exhibited greater improvements than the control group in effective activation of reading strategies, such as taking notes while reading. They also showed greater improvement in writing strategies—especially those related to planning their texts—considering structural aspects of the text when planning, such as the introduction and conclusion, and explicitly demonstrating the use of strategies. The greater improvement in the experimental group’s text quality was maintained over the long term, shown by the follow-up assessment one year later, although it is worth emphasizing that both groups demonstrated improvements in text product scores at the follow-up evaluation. This shows some consolidation of synthesis competencies in university students, presumably driven by their progress in their degree courses. These are courses that generally use active methodologies requiring increasingly active, strategic, and self-regulated learning from the student (Robledo et al., 2015; García et al., 2017).

Hence, in light of the scientific literature in this field, our study confirms that—with proper guidance from teachers via implementing strategic instructional processes (Graham et al., 2018; Graham and Harris, 2018) within the context of the curriculum study plan (Zhang, 2013)—students can adapt and re-work their own strategies (Butler, 1994, 1995, 1996, 1997, 1998), in this case for reading and writing, and apply them in a self-regulated manner when doing complex academic tasks, such as synthesis, improving the quality of their texts and of some of the cognitive processes involved (Boscolo et al., 2007; Mateos et al., 2018; Granado-Peinado et al., 2019). Despite that, the program implemented in this study did not contribute to any significant improvements in the processes of selecting information required by ST and related to selecting ideas from source texts. These processes seem to be among the most well-developed in university students (Robledo et al., 2018), which is why the range of improvement is smaller. The program also failed to produce significant improvements in processes for connecting information, including cohesion or incorporating ideas from source texts into the ST. These processes for connecting information require students to deeply read the source texts in combination, which in this case are complex academic texts. They also involve rewriting and connecting the ideas taken from the texts into a new written discourse. This may represent an additional challenge for students who, faced with the complexity of the materials they have to read, only engage with them superficially and fail to achieve the in-depth understanding that would allow them to write about the new ideas (Beck et al., 1996; van Ockenburg et al., 2019). Processes related to reviewing writing also failed to show any significant improvement, possibly because students activated them when they were cognitively drained after having focused their efforts on detailed planning or even after drafting the text. Students need greater cognitive flexibility and to recursively activate all of the cognitive processes involved in producing STs to achieve optimum levels of functionality (Mateos et al., 2008). Finally, the lack of a proper metacognitive representation of synthesis tasks often conceptualized by students as summaries (Mateos and Solé, 2009; Robledo et al., 2018), may also explain the poor activation of some core processes for these tasks. In any case, it is clear that our results call for future studies that seek deeper explanations and that can provide more specific guidance to teachers in terms of functional activation of all of the processes involved in producing synthesis texts through effective development and deployment of specific strategies linked to each process and through spending more time on their practical application (Merrill, 2002; Van Ockenburg et al., 2019).

Despite that, we can conclude that instruction based on stimulating students’ own strategies and encouraging self-regulated application of them in synthesis tasks is effective and contributes to students producing better quality texts, organizing information better, and more effectively activating some of the reading and writing processes these types of tasks call for. This may be because this type of instruction allows students more control over their cognitive and emotional processes, meaning that they approach these tasks with greater motivation and are able to keep at them and complete them successfully, even with the challenges they present (Butler, 1998; Navea-Martín and Suárez-Riveiro, 2017; van Ockenburg et al., 2019). To sum up from a theoretical-empirical perspective, we can conclude that implementation of strategic instruction programs aimed at encouraging ST mastery at the university level should consider how strategically mature the students are and their potential to create and modify their own strategies (Kieft et al., 2007; Robledo et al., 2018; Arias-Gundín and Robledo, 2023). Teachers must focus on guiding and prompting students so that they develop and implement their own strategies in a self-regulated manner when doing ST. Therefore, it seems that in the university context, sequences of strategic instruction programs can be simplified, as can their component parts. Students, with the teacher’s guidance, can develop their own strategies and apply them appropriately to perform synthesis tasks.

Nonetheless, these conclusions should be understood considering the limitations of the study. One limitation is the small sample size, which was taken from a specific course that was part of one degree at one university. This limits the possibilities of generalizing the results, and means that it would be interesting for future studies to use a larger sample and also to do more complex statistical analysis, such as mixed-effects models, with observations grouped within individual students and students nested within classes, given that the present study is not immune to the clustering effect. This was an early pilot study in this field, which used a similar sized sample to other international studies in the same area (reviewed in the introduction). In addition, having 20–25 students in a class was ideal for learning. Given that, more studies, and more robust statistical analyses, are needed to test whether synthesis writing instruction is effective for a wider range of students. It would also be useful to consider other types of intrinsic student variables that might affect the results. In this case, because the instruction was based on the students readjusting their own strategies, it would be useful to determine a general strategic profile for the students and a profile of their reading and writing strategies, which seem to vary between students and affect how they face learning activities in general and written tasks in particular (Kieft et al., 2007; van der Loo et al., 2018; Arias-Gundín and Robledo, 2023). In addition, it would be interesting to use more evaluation instruments, including ones which would allow measurement of qualitative changes in critical writing skills, which would guide interventions more accurately (Deane and Song, 2014). Another limitation to highlight is that the teachers tasked with implementing the program had experience in teaching written composition. This makes it difficult to say whether other teachers with less expertise in teaching processes would be able to apply the program with similar success. In line with this, on a practical note, it is worth emphasizing that the instructional program tested in this study could be applied in different subjects and may be an interesting methodological tool for teachers because, in addition to helping improve students’ syntheses, it provides an active method to access content for various learning goals (van Ockenburg et al., 2019), facilitating students’ autonomous learning and skill development.

## Data availability statement

The raw data supporting the conclusions of this article will be made available by the authors, without undue reservation.

## Ethics statement

The studies involving humans were approved by Comité de ética University of León for the studies involving humans. The studies were conducted in accordance with the local legislation and institutional requirements. All students agreed to participate voluntarily after verbal informed consent was obtained. The studies were conducted in accordance with the local legislation and institutional requirements. The participants provided their written informed consent to participate in this study.

## Author contributions

PR: Conceptualization, Data curation, Formal analysis, Funding acquisition, Investigation, Methodology, Project administration, Resources, Software, Supervision, Validation, Visualization, Writing – original draft, Writing – review & editing. MÁ: Conceptualization, Funding acquisition, Resources, Writing – review & editing.
